# UHRF1 depletion sensitizes retinoblastoma cells to chemotherapeutic drugs via downregulation of XRCC4

**DOI:** 10.1038/s41419-017-0203-4

**Published:** 2018-02-07

**Authors:** Heng He, Chunsik Lee, Jong Kyong Kim

**Affiliations:** State Key Laboratory of Ophthalmology, Zhongshan Ophthalmic Center, Sun Yat-sen University, Guangzhou, 510060 China

## Abstract

UHRF1 (ubiquitin-like with PHD and ring finger domains 1) is highly expressed in various human cancers including retinoblastoma, and associated with tumor-promoting effects such as inhibition of apoptosis and high proliferation. However, the molecular mechanisms underlying tumor-promoting functions of UHRF1 in retinoblastoma still remain elusive. Here, we show that stable knockdown of UHRF1 renders retinoblastoma cells sensitized to conventional chemotherapeutic drugs such as etoposide and camptothecin, resulting in enhanced DNA damage and apoptotic cell death. We found that UHRF1-depleted retinoblastoma cells can recognize DNA damages normally but have markedly low expression of XRCC4 (X-ray repair cross complementing 4) among the components of nonhomologous end-joining (NHEJ) repair complex. Conversely, overexpression of UHRF1 increased the XRCC4 expression and stable knockdown of XRCC4 sensitized retinoblastoma cells to etoposide treatment, suggesting that XRCC4 is a key mediator for the drug sensitivity upon UHRF1 depletion in retinoblastoma cells. Consistent with the findings, chromatin association of DNA ligase IV in response to acute DNA damage was found to be significantly reduced in UHRF1-depleted retinoblastoma cells and functional complementation for XRCC4 in UHRF1-depleted cells attenuated the drug sensitivity, demonstrating that XRCC4 downregulation in UHRF1-depleted cells impaired DNA repair and consequently induced robust apoptosis upon genotoxic drug treatment. In human primary retinoblastoma, high expression of UHRF1 and XRCC4 could be detected, and elevated XRCC4 expression correlated with reduced apoptosis markers, implying that UHRF1-mediated XRCC4 upregulation under pathophysiological conditions triggered by *RB1* gene inactivation may confer protection against endogenous DNA damages that arise during retinoblastoma development. Taken together, these results present a new mechanistic insight into how UHRF1 mediates its tumor-promoting functions in retinoblastoma, and also provide a basis for UHRF1 targeting to improve the efficacy of current chemotherapy for retinoblastoma treatment.

## Introduction

Retinoblastoma is a childhood malignancy initiated by *RB1* gene mutations in the developing retina^1^. Among the numerous treatment options for retinoblastoma, chemotherapy has been an important therapeutic modality for preserving the eye and vision^2,3^. However, the efficacy of chemotherapy is often limited by development of drug resistance and adverse side effects. Identification of new genetic pathways or druggable molecular targets that may sensitize retinoblastoma cells to standard chemotherapeutic regimens may provide an attractive strategy to increase the efficacy of the current chemotherapy.

UHRF1 (ubiquitin-like with PHD and RING finger domains 1) is highly expressed in various cancer cells and its overexpression has been associated with tumor-promoting effects represented by high proliferative potential and inhibition of apoptosis^4,5^. These tumor-promoting functions of UHRF1 are known to be mediated by both epigenetic and non-epigenetic mechanisms^6,7^. An early study reported that mouse *Uhrf1* (Np95)-null embryonic stem (ES) cells are more sensitive to genotoxic stress including irradiation and DNA damaging agents than *Uhrf1*^+/+^ and *Uhrf1*^+/−^ ES cells, suggesting a role for UHRF1 in cellular response pathways against DNA damage and replication block^8^. Subsequently, human UHRF1 was also shown to have similar functions to those of mouse UHRF1 as down-modulation of the protein in human somatic cells increased the sensitivity to diverse genotoxic insults^9^. Consistent with these earlier findings, overexpression or downregulation of UHRF1 in various cancer cell lines decreased or increased the radiosensitivity^10–13^. In addition, UHRF1 depletion was reported to affect the genome stability as demonstrated by a higher frequency of sister chromatid exchange and chromosome aberrations in *Uhrf1*-/- ES cells and UHRF1-knockdown HeLa cells^8,12^. Of note, UHRF1-depleted HCT116 cells activated DNA damage responses without any extrinsic DNA damages and induced G2/M cell cycle arrest followed by apoptosis^14^. Subsequent studies revealed that UHRF1 participates in the recognition and repair of DNA interstrand crosslink lesions, providing evidences that UHRF1 can physically sense the DNA lesions and recruit relevant nuclear factors required for the repair^15,16^. Interestingly, a recent study demonstrated that UHRF1 contributes to cell cycle-dependent repair choices for DNA double-strand breaks (DSB) by employing its E3 ubiquitin ligase activity for RIF1 repair factor and consequently favoring homologous recombination in S phase^17^. Collectively, these studies support the notion that UHRF1 is involved in genome maintenance, DNA damage response, and repair. As these biological processes can directly affect cell survival and death^18,19^, UHRF1 targeting may be exploited to improve the current cancer therapy.

As in other human cancers, UHRF1 is highly expressed in retinoblastoma and exerts tumor-promoting effects as demonstrated by impaired colony formation and reduced size of xenografted tumors upon UHRF1 down-modulation in retinoblastoma cells^20^. Although UHRF1 has well-established roles in DNA methylation as an epigenetic regulator implicated in tumor development^6^, our comprehensive DNA methylome analyses revealed that the tumor-promoting functions of UHRF1 in retinoblastoma are largely independent of its role in DNA methylation, suggestive of other mechanisms involved in retinoblastoma development^21^. Given the evidences documented on the potential roles of UHRF1 in maintenance of genome integrity and DNA repair, we explored a possibility that high UHRF1 expression in retinoblastoma may contribute to protection against endogenous DNA damages and genotoxic drugs to promote the tumor cell survival and growth, and investigated the functions of UHRF1 in response to DNA damages induced by chemotherapeutic drugs in retinoblastoma cells.

## Results

### UHRF1 depletion sensitizes retinoblastoma cells to chemotherapeutic drugs

**Fig. 1:**
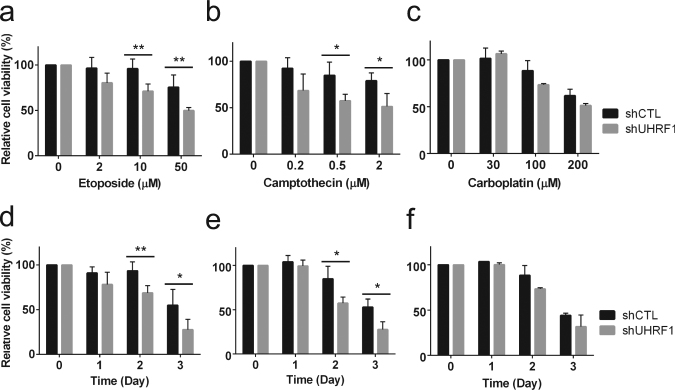
UHRF1 depletion sensitizes retinoblastoma cells to chemotherapeutic drugs. **a**–**c** Dose–response study showing the relative sensitivity to drugs. Stable control-knockdown (shCTL) and UHRF1-knockdown (shUHRF1) Y79 cells were exposed to various concentrations of chemotherapeutic drugs for 48 h, as indicated. **d**–**f** Time-course study showing the relative sensitivity to drugs. Cells were treated with 10 µM etoposide (**d**), 0.5 µM camptothecin (**e**), and 100 µM carboplatin (**f**) for the indicated time. The results at each data point are shown as the mean ± standard deviation (SD) of % fold changes from three independent experiments, relative to the cell viability in untreated group. *P < 0.05, **P < 0.01

In an attempt to investigate how UHRF1 contributes to retinoblastoma development, we tested a possibility that high UHRF1 expression in retinoblastoma cells may endow the cells with the resistance against chemotherapeutic drugs used for retinoblastoma treatment in clinical settings. Etoposide (topoisomerase II inhibitor), camptothecin (topoisomerase I inhibitor), and carboplatin were used for dose–response and time-course studies on control and UHRF1-knockdown Y79 retinoblastoma cells (Fig. 1). UHRF1-depleted retinoblastoma cells showed a clear increase in sensitivity to the two topoisomerase inhibitors, whereas only modest increase in sensitivity to carboplatin was observed at high doses or longer treatment.

### Enhanced drug sensitivity upon UHRF1 depletion involves apoptotic cell death

**Fig. 2:**
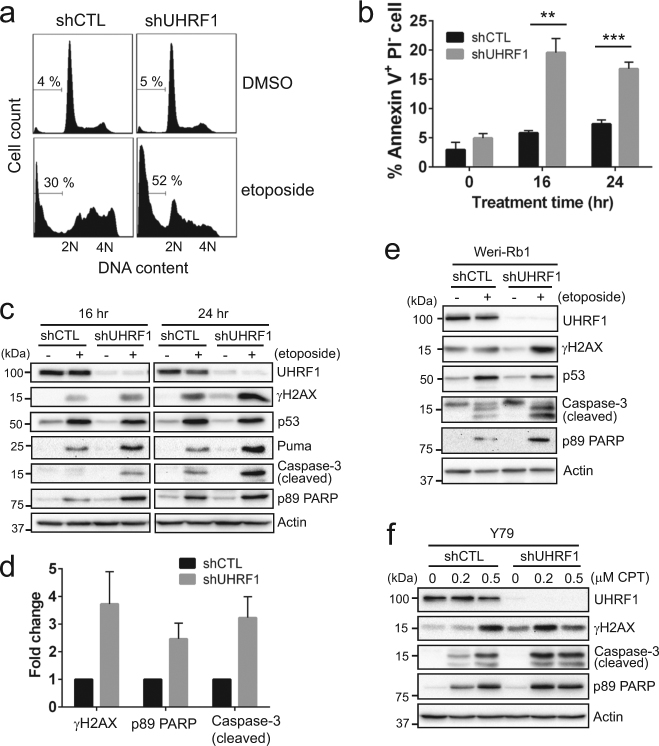
Enhanced drug sensitivity upon UHRF1 depletion involves apoptotic cell death. **a** Sub-G1 population detected by flow cytometry in control (shCTL) and UHRF1-knockdown (shUHRF1) Y79 cells treated with vehicle or 10 µM etoposide for 24 h. The percentage of sub-G1 population is shown. **b** Quantification of early apoptotic cell death by Annexin V-PI staining. Control and UHRF1-knockdown Y79 cells were treated with 10 µM etoposide for the time indicated. The data represent the mean ± SD of % Annexin V^+^ PI^−^ population from triplicate experiments. **P < 0.01, ***P < 0.001. **c** Immunoblots for indicated proteins in Y79 shCTL and shUHRF1 cells after exposure to 10 µM etoposide for 16 h and 24 h. **d** Densitometric analyses of the indicated proteins for 24 h-treatment in **c**. The graph is shown as the mean ± SD of fold changes from four independent experiments, relative to the normalized level in shCTL cells. **e** Immunoblots for indicated proteins in Weri-Rb1 shCTL and shUHRF1 cells treated for 24 h. **f** Expression of indicated proteins in UHRF1-knockdown Y79 cells after treatment with various concentrations of camptothecin (CPT) for 24 h

To examine if the increased drug sensitivity upon UHRF1 depletion accompanies higher apoptotic responses, Y79 knockdown cells were treated with etoposide and analyzed for several apoptosis markers. Sub-G1 and annexin V^+^ PI^−^ early apoptotic populations were higher in UHRF1-depleted cells than in control cells (Fig. 2a, b). Upon etoposide treatment, both control and UHRF1-knockdown cells showed p53 induction and the increase in phosphorylation of upstream kinases in DNA damage responses (Supplementary Fig. S1). However, UHRF1-depleted cells displayed more robust apoptotic responses demonstrated by elevated levels of cleaved caspase-3 and poly(ADP-ribose) polymerase (PARP) (Fig. 2c, d). Interestingly, higher phosphorylation of histone H2AX at Ser139 (γH2AX) was detected in UHRF1-depleted cells, suggestive of higher susceptibility to DSB inducers such as etoposide. The higher sensitivity to etoposide was not due to the changes in topoisomerase expression in UHRF1-depleted cells (Supplementary Fig. S2). Similar apoptotic responses upon UHRF1 knockdown were observed for another retinoblastoma cell line, Weri-Rb1 (Fig. 2e). Consistent with the results shown in Fig. 1b, e, UHRF1-depleted Y79 cells also exhibited higher apoptosis in response to camptothecin (Fig. 2f).

### UHRF1 depletion does not impair DNA damage sensing

**Fig. 3:**
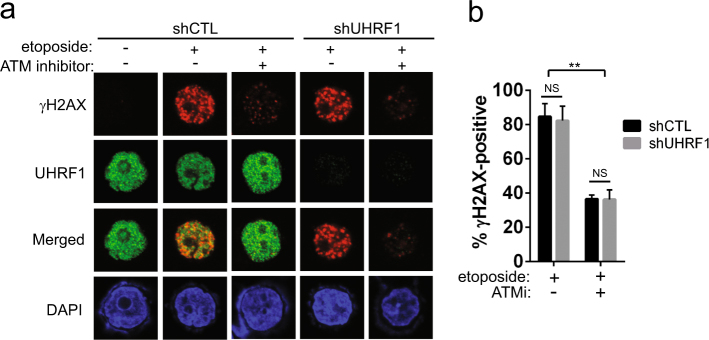
UHRF1 depletion does not impair DNA damage sensing. **a** Immunostaining for γH2AX as an indicator for DNA damage. Y79 shCTL and shUHRF1 cells were pre-treated with either vehicle or 10 µM ATM inhibitor (ATMi) for 2 h, followed by the treatment with 10 µM etoposide for 2 h. **b** Quantification of γH2AX-positive cells shown in **a**. Over 300 total cells per group were evaluated for γH2AX-positivity (>10 foci/cell), and the data represent the mean ± SD from three independent experiments. **P < 0.01, NS not significant

Several studies showed that UHRF1 is recruited to DNA damage sites and required for DNA damage recognition in response to irradiation and DNA interstrand crosslinks in various cancer cells^12,15,16^. As higher levels of γH2AX were detected in UHRF1-knockdown cells following the prolonged etoposide treatment for 24 h (Fig. 2), we suspected that the observation in other cancer cell lines may not hold true for retinoblastoma cells. To verify our prediction, we examined the distribution pattern and number of γH2AX foci in UHRF1-depleted cells in comparison with control cells after a short treatment with etoposide (Fig. 3). UHRF1-depleted cells exhibited a similar level of γH2AX foci compared to their control counterparts, and broad distribution of UHRF1 in cell nuclei indicated that UHRF1 was not specifically recruited to DNA damage sites marked by γH2AX (Fig. 3a, b). Furthermore, γH2AX foci formation was similarly inhibited by an inhibitor of ATM, one of the kinases responsible for H2AX phosphorylation upon DNA damage. These data imply that UHRF1 is not required for DNA damage recognition in retinoblastoma cells and may play other roles in DNA damage responses against etoposide treatment. Considering the higher levels of γH2AX detected in UHRF1-knockdown cells following the longer etoposide treatment (Fig. 2c), these results suggest that DNA repair in UHRF1-depleted cells may not be as efficient as in control cells, leaving more unrepaired DNA lesions, which would result in higher γH2AX levels and apoptosis.

### XRCC4 downregulation in UHRF1-depleted retinoblastoma cells impedes recovery from DNA damage

**Fig. 4:**
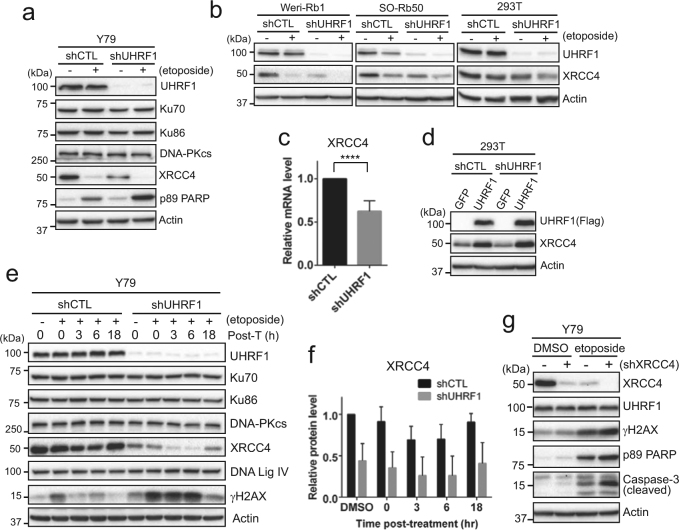
XRCC4 downregulation in UHRF1-depleted retinoblastoma cells impedes recovery from DNA damage. **a** Immunoblots for indicated proteins in shCTL and shUHRF1 Y79 cells. Cells were treated with 10 µM etoposide for 24 h. **b** Expression of XRCC4 in UHRF1-depleted Weri-Rb1, SO-Rb50, and 293T cells after treatment with 10 µM etoposide for 24 h. **c** qRT-PCR analysis of relative XRCC4 expression in Y79 shCTL and shUHRF1 cells. The bar graph is shown as the mean ± SD of fold changes from five independent experiments, relative to the normalized XRCC4 expression in control-knockdown cell. ****P < 0.0001. **d** XRCC4 expression after adenoviral expression of either GFP or Flag-tagged UHRF1 in shCTL and shUHRF1 293 T cells. **e** Immunoblots for indicated proteins showing the recovery kinetics from acute DNA damage induced by etoposide. Cells were treated with 10 µM etoposide for 1 h, and then placed in fresh media without drugs for the indicated time post-treatment (post-T). **f** Densitometric analysis of XRCC4 protein levels in **e**. The graph is shown as the mean ± SD of fold changes from five independent experiments, relative to the normalized level in DMSO-treated shCTL Y79 cells. **g** Immunoblots for indicated proteins in shCTL (−) and shXRCC4 (+, clone #40116) Y79 cells after treatment with vehicle or 10 µM etoposide for 24 h

As DNA damage induced by topoisomerase II inhibition is known to be repaired by NHEJ repair^22,23^, we examined several factors involved in NHEJ repair in UHRF1-depleted Y79 cells (Fig. 4a). XRCC4 (X-ray repair cross complementing 4) was shown to be markedly low in untreated UHRF1-knockdown cells compared to their control counterparts, whereas other NHEJ factors did not display any noticeable changes upon UHRF1 knockdown. Etoposide treatment for 24 h reduced the XRCC4 level in both cell groups, supporting the previous report that XRCC4 is a target for apoptotic proteases in response to DNA damage^24^. The XRCC4 downregulation upon UHRF1 knockdown was observed in other retinoblastoma cell lines (Weri-Rb1 and SO-Rb50) and 293T cells (Fig. 4b), and occurred at the transcript level (Fig. 4c). Adenoviral overexpression of UHRF1 increased the XRCC4 protein level in both control and UHRF1-depleted cells (Fig. 4d), indicating that XRCC4 expression may be regulated by UHRF1. As UHRF1 is an E3 ligase that can regulate protein stability and signaling by ubiquitinating its target proteins^7,17,25^, we examined the stability of XRCC4 protein in UHRF1-depleted cells by cycloheximide chase. Our results showed that XRCC4 is a relatively stable protein and there is no significant difference in turnover kinetics between control and UHRF1-depleted Y79 cells other than the reduced basal XRCC4 level upon UHRF1 depletion (Supplementary Fig. S3). Furthermore, in vivo ubiquitination assay revealed that ubiquitination of XRCC4 is similar between control and UHRF1-depleted cells in the presence and absence of MG132 and etoposide (Supplementary Fig. S4), demonstrating that UHRF1 does not regulate ubiquitination of XRCC4.

To test if the higher level of γH2AX in UHRF1-depleted cells is associated with impaired DNA repair, control and UHRF1-knockdown Y79 cells were acutely exposed to etoposide and monitored for recovery from the acute DNA damage based on the kinetics of induction and resolution of the γH2AX signal post-treatment. UHRF1-depleted cells exhibited more robust induction of γH2AX in response to the acute DNA damage than control cells, and the high γH2AX level sustained longer in UHRF1-knockdown cells, whereas control cells resolved the signal within 3 h post-treatment (Fig. 4e). Similarly, kinetics of γH2AX foci resolution determined by immunofluorescence after the acute DNA damage also revealed that UHRF1 depletion impedes recovery from DNA damage (Supplementary Fig. S5), suggesting that UHRF1-depleted cells may have impaired DNA repair. Among the NHEJ factors, only XRCC4 was downregulated in UHRF1-knockdown cells (Fig. 4e), and the XRCC4 recovery kinetics after the acute DNA damage appeared to be similar between the two cell groups (Fig. 4f). The direct knockdown of XRCC4 in Y79 cells followed by etoposide treatment induced higher apoptotic responses than in control cells, verifying that the robust apoptosis in UHRF1-depleted cells upon DNA damage is indeed associated with the XRCC4 downregulation (Fig. 4g and Supplementary Fig. S6). Consistent with these findings, inhibition of DNA-PK in UHRF1-knockdown cells generated additive effects on apoptosis in response to etoposide treatment (Supplementary Fig. S7).

### XRCC4 downregulation in UHRF1-depleted cells impairs DNA repair and increases drug sensitivity

**Fig. 5:**
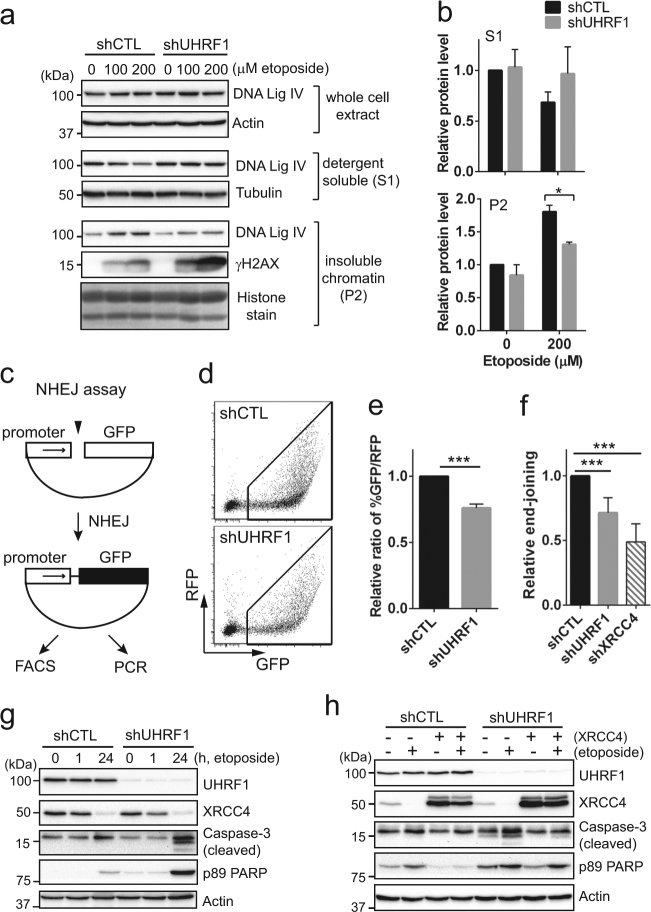
XRCC4 downregulation in UHRF1-depleted cells impairs DNA repair and increases drug sensitivity. **a** DNA ligase IV recruitment onto chromatin upon acute DNA damage. Y79 shCTL and shUHRF1 cells were exposed to high doses of etoposide for 50 min and subjected to immediate cell fractionation. Whole-cell lysates, detergent-soluble (S1) cytosolic fraction, and detergent-insoluble (P2) chromatin fraction were analyzed by immunoblots for the indicated proteins. Tubulin and histones were used as a loading control for cytosolic and chromatin extracts, respectively. **b** Densitometric analysis for the DNA ligase IV level in cytosolic and chromatin fractions shown in **a**. The data represent the mean ± SD of fold changes from three independent experiments, relative to the normalized DNA ligase IV level in DMSO-treated shCTL Y79 cells. *P < 0.05. **c** Schematic of plasmid-based NHEJ assay. **d**,** e** Representative image and computed data for fluorescence-activated cell sorting (FACS)-based NHEJ efficiency assay. GFP expression driven by NHEJ repair was normalized by the expression of intact RFP transfection control. The data represent the mean ± SD of fold changes from three independent experiments, relative to the normalized ratio of %GFP/%RFP in shCTL 293 T cells. ***P < 0.0001. **f** Relative end-joining efficiency determined by qPCR and shown as the mean ± SD of fold changes relative to shCTL cells from three independent experiments. Abundance of re-joined plasmid junctions was normalized by uncut GFP-coding sequence. Relative end-joining efficiency in shXRCC4 293T cells is shown as a positive control. ***P < 0.0001. **g** Increased apoptosis in UHRF1-depleted 293T cells in response to etoposide (10 µM). **h** Attenuated apoptotic responses to etoposide treatment upon XRCC4 overexpression. At 24 h post-adenoviral XRCC4 transduction in shCTL and shUHRF1 293T cells, treatment with either vehicle or 10 µM etoposide was performed for 24 h

To examine if the reduced XRCC4 level is associated with impaired DNA repair efficiency in UHRF1-depleted Y79 cells, we first investigated whether DNA ligase IV can be normally loaded onto the damaged chromatin in the UHRF1-knockdown cells as XRCC4 is required for activation and stabilization of its binding partner DNA Ligase IV^26,27^. Control and UHRF1-knockdown Y79 cells were acutely exposed to high doses of etoposide to increase the intensity of DNA damage, and immediately subjected to detergent-based cell fractionation. While DNA ligase IV levels were similar in whole-cell extracts, detergent-insoluble chromatin fractions of UHRF1-knockdown cells showed lower DNA ligase IV association in response to acute DNA damage than those of control cells (Fig. 5a, b). We also examined the chromatin association of other NHEJ factors in UHRF1-depleted Y79 cells, but did not detect much difference compared to the case of control-knockdown cells (Supplementary Fig. S8). Next, we tested if UHRF1 depletion would indeed reduce the NHEJ repair efficiency by a plasmid-based assay (Fig. 5c). Compared to control cells, UHRF1-depleted cells showed a modest but significant decrease in the end-joining efficiency by two different detection methods (Fig. 5d–f). As in retinoblastoma cells, UHRF1 knockdown in 293T cells induced more intense apoptosis upon etoposide treatment (Fig. 5g), and overexpression of XRCC4 in UHRF1-depleted cells attenuated the apoptosis (Fig. 5h). Altogether, these results suggest that XRCC4 downregulation is a mechanism underlying the increased sensitivity to DSB inducers in UHRF1-depleted cells by impairing DNA repair.

### XRCC4 expression in human retinoblastoma

**Fig. 6:**
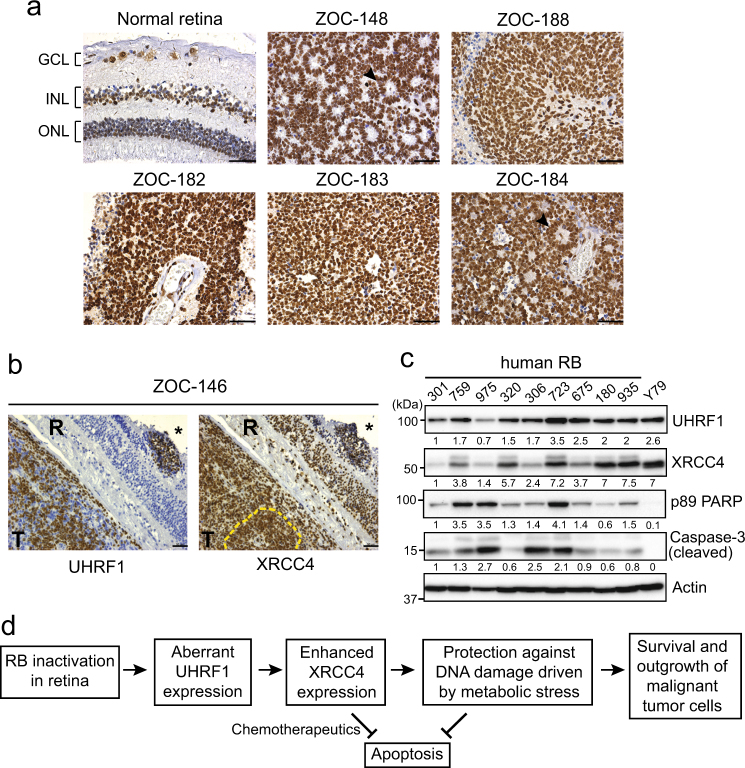
XRCC4 expression in human primary retinoblastoma. **a** Immunostaining of XRCC4 in human retinoblastoma (*n* = 10) and normal retina (42 years of age) sections. Nuclei were counterstained with hematoxylin. Black arrowheads in ZOC-148 and ZOC-184 indicate rosettes characteristic of differentiated retinoblastoma. GCL ganglion cell layer, INL inner nuclear layer, ONL outer nuclear layer, Scale bar: 50 µm. **b** Expression of UHRF1 and XRCC4 in human retinoblastoma. Two serial sections of ZOC-146 immunostained for UHRF1 and XRCC4 are shown as representative images. Dense staining of XRCC4 is visible in tumor foci in the retina (marked by *) and vitreous tumor region (marked by yellow dashed lines) where UHRF1 is highly expressed. T tumor, R retina, scale bar: 50 µm. **c** Immunoblots for indicated proteins in human retinoblastoma (RB) lysates. Relative abundance of proteins determined by densitometry is shown below each panel. **d** Proposed model of UHRF1-mediated tumor promotion in retinoblastoma

As UHRF1 is highly expressed in human retinoblastoma^21^, we examined the expression of XRCC4 in human retinoblastoma in comparison with normal retina. Unlike UHRF1, XRCC4 is expressed in both nuclear layers and ganglion cells of normal retina at detectable levels (Fig. 6a), supporting that XRCC4 is essential for normal genome maintenance^28^. However, the XRCC4 expression in human retinoblastoma was much higher than that of normal retina (Fig. 6a). To compare the expression patterns of UHRF1 and XRCC4, we used two serial retinoblastoma sections for UHRF1 and XRCC4 immunostaining. For most sections with high tumor cell density, both UHRF1 and XRCC4 are highly expressed in an indistinguishable manner (Supplementary Fig. S9). However, tumor tissues with relatively intact retinal structure showed denser XRCC4 staining in the regions where UHRF1 is highly expressed (Fig. 6b), implying that UHRF1 expressed during tumorigenesis of retina may further promote the XRCC4 expression. As immunostaining does not allow for a collective analysis for the whole tumor tissues, we used fresh tumor lysates to examine the expression level of UHRF1, XRCC4, and a few apoptosis markers (Fig. 6c). Apart from one sample (#723), high levels of XRCC4 in human retinoblastoma moderately correlated with lower levels of apoptosis markers. This suggests that the highly expressed XRCC4 may protect tumor cells from apoptosis when endogenous DNA damages are generated by their excessive proliferation (Fig. 6d).

## Discussion

UHRF1 has been gaining attention in cancer research not only as an important epigenetic regulator for DNA methylation and histone modifications but also as a critical regulator for cancer cell proliferation and survival^4,5^. As in many human cancers, retinoblastoma has a high level of UHRF1 expression that contributes to retinoblastoma development^20^. In this study, we demonstrate that high UHRF1 expression in retinoblastoma promotes tumor cell survival against genotoxic insults by enhancing DNA repair and consequently reducing apoptotic cell death.

Since the first study reporting potential roles for UHRF1 in maintenance of genomic stability in response to DNA damage and replication block in mouse *Uhrf1*^-/-^ ES cells^8^, further evidences supporting the functions of UHRF1 in DNA repair and genome maintenance have become available. One mechanism accounting for these functions is that UHRF1 can directly participate in DNA repair by recognizing DNA lesions and recruiting relevant repair factors as demonstrated in the case of DNA interstrand crosslink repair^15,16^. However, our results revealed that UHRF1 depletion in retinoblastoma cells does not affect the recognition of DNA damages generated by etoposide treatment, and UHRF1 does not appear to be recruited to DSBs marked by γH2AX. The difference may be associated with using different cell types and genotoxic agents, but points out that UHRF1 employs a different mechanism in retinoblastoma cells to promote cell survival against the potent DSB inducer. Another plausible mechanism that may explain the role of UHRF1 in DNA repair and genome maintenance is regulation of repair factor abundance and availability. In fact, expression of several NHEJ repair factors in response to irradiation was shown to be affected by the UHRF1 status in cells although the functional relationship between the repair factors and UHRF1 remained unclear^10,11,13^. In this regard, we examined nuclear factors involved in the repair of etoposide-induced DSBs, and identified XRCC4 as a key mediator regulated by UHRF1 in retinoblastoma cells. Our data demonstrate that UHRF1-mediated XRCC4 upregulation occurs at the transcript level in retinoblastoma cells, and the enhanced XRCC4 expression reduces etoposide-induced apoptosis by ensuring efficient chromatin loading of DNA ligase IV for the end-joining activity required for the repair following the genotoxic insults. Although UHRF1 has an E3 ligase activity and ubiquitination-mediated signaling has important implications in DNA damage responses and repair^17^, we did not find any experimental evidences for alterations in stability and ubiquitination patterns of XRCC4 protein upon UHRF1 depletion, implying that XRCC4 is not a target for the UHRF1 E3 ligase activity. Instead, a recent study reported that SCF^FBXW7^ E3 ligase is responsible for XRCC4 ubiquitination to facilitate NHEJ repair against ionizing radiation^29^. Currently, how UHRF1 increases the XRCC4 transcription is unclear, however, the transcriptional effect may be indirectly mediated via other UHRF1 target(s) because UHRF1 works mostly as a transcriptional repressor by recruiting repressive chromatin modifiers onto the target promoters^30,31^.

Of note, XRCC4 expression is detectable in human normal retina unlike the case of UHRF1, and our data suggest that high UHRF1 expression during retinoblastoma tumorigenesis may further augment the XRCC4 expression in tumors. This finding may explain why UHRF1 depletion in retinoblastoma cells only attenuates but does not abrogate the XRCC4 expression, suggesting that the basal level of XRCC4 expression required for normal genome maintenance is mediated by other factors rather than UHRF1. The elevated XRCC4 expression under pathophysiological conditions triggered by *RB1* gene inactivation and subsequent induction of UHRF1 expression in the developing retina may promote survival and outgrowth of malignant tumor cells by increasing the repair efficiency against endogenous genotoxic stress that may arise during tumorigenesis (Fig. 6d). As oxidative stress even at low levels was shown to induce DSBs^32^ and NHEJ repair-deficient cells were found to be hypersensitive to oxidative stress and consequently undergo apoptosis^33^, it is plausible that highly proliferating retinoblastoma cells would encounter endogenous metabolic stress such as reactive oxygen species and the enhanced repair would endow the tumor cells with a selective advantage to evade apoptosis. As human retinoblastoma has intact *TP53* gene and functional p53 pathway^34,35^, how retinoblastoma cells cope with p53-mediated tumor surveillance and avoid apoptosis has been a question of great interest. In this context, MDM4 was found to suppress p53-mediated apoptosis during tumor progression^36^, and MDM2 was shown to promote retinoblastoma cell proliferation by upregulating MYCN translation independently of p53, proposing that high MDM2 expression in retinoblastoma may eliminate a need for genetic mutations in the p53 pathway^37^. Our study may provide a novel alternative mechanism that can alleviate p53-mediated apoptosis in retinoblastoma. Following DNA damage, the intact p53 pathway in retinoblastoma cells would launch normal DNA damage responses that would initiate cell cycle arrest and DNA repair before the balance is completely shifted to pro-death pathways depending on the severity of DNA damage. During the initial pro-survival signaling, the enhanced repair capacity by XRCC4 upregulation in retinoblastoma cells would reduce p53-mediated apoptosis and may even generate new genetic mutations conducive to tumor cell survival due to the error-prone nature of NHEJ repair.

Decision between cell survival and death following genotoxic insults plays critical roles in tumor initiation and progression, and also in determining the efficacy of antitumor therapy^18,19^. Although the efficacy of chemotherapy with genotoxic drugs is often limited by development of drug resistance and adverse side effects, chemotherapy has been an important therapeutic modality for retinoblastoma treatment as an efficient way to reduce the size of tumors by systemic administration of drugs, and also as a regional treatment option directly towards the eye^2,3^. Therefore, identification of new genetic pathways or molecular targets whose disruption may sensitize retinoblastoma cells to standard chemotherapeutics would be beneficial to improve the efficacy of current chemotherapy for retinoblastoma treatment. In this sense, UHRF1 may serve as an attractive therapeutic target as it fits reasonably well with the rationale above. First, UHRF1 is not expressed in normal retina, which confers selectivity to UHRF1 targeting. Second, UHRF1 down-modulation in retinoblastoma cells exerts a minor effect on DNA methylome^21^, implying that local UHRF1 targeting in retinoblastoma may be safe and efficacious without complications involving DNA methylation. Third, UHRF1 down-modulation sensitizes retinoblastoma cells to standard chemotherapeutic drugs.

In summary, we present a novel mechanism for tumor-promoting functions of UHRF1 in retinoblastoma and provide an additional insight into how retinoblastoma cells may alleviate p53-mediated tumor suppression, with potential implications of UHRF1 targeting as an efficient strategy to improve the efficacy of current chemotherapy for retinoblastoma treatment.

## Materials and methods

### Cell culture and human tissues

Y79, Weri-Rb1, and 293T cells were obtained from American Type Culture Collection (ATCC), and SO-Rb50 was established at the Zhongshan Ophthalmic Center (ZOC)^38^. All retinoblastoma cell lines were maintained in RPMI1640 containing 10% FBS and penicillin–streptomycin (Gibco). Etoposide and camptothecin were purchased from Sigma and dissolved in dimethyl sulfoxide (DMSO) at a concentration of 50 and 20 mM, respectively. Carboplatin (Sigma) was prepared in sterile water (25 mM), and ATM inhibitor (KU-55933, Selleckchem) and DNA-PK inhibitor (KU-57788, Selleckchem) were dissolved in DMSO (50 and 7.25 mM, respectively). Cells were treated with drugs to final concentrations in culture media with vehicle controls set up in parallel. Stable UHRF1-knockdown cells were generated by lentiviral shRNA transduction as described previously^21^. Lentiviral knockdown of XRCC4 was performed using two shRNA clones from Dharmacon (TRCN0000040114, TRCN0000040116). Adenoviruses for UHRF1 (VH819327) and XRCC4 (VH898008) expression were purchased from Vigene Biosciences. After 24 h post-adenoviral transduction, cells were subjected to drug treatment. Human normal retina and primary retinoblastoma tissues were obtained from the eye bank, ocular tumor division, and department of pathology at the ZOC. The study with human clinical samples was approved by the ZOC institutional review board. All human specimens used for this study were de-identified, and informed consent forms were obtained.

### Cell viability and apoptosis assays

Y79 cells were plated in 96-wells at a density of 5 × 10^3^ cells/well in four replicates per treatment group. The next day, cells were treated with drugs at indicated concentrations and cell viability was determined by MTT cell proliferation assay (Roche) following the manufacturer’s instruction. For apoptosis assay, cells were harvested by taking all suspension cells in the medium and apoptotic cell populations were detected by flow cytometry using the Annexin V-FITC Apoptosis Kit (Roche) according to the manufacturer’s instruction.

### Western blot

Cleared lysates (25–30 µg) were subjected to 7.5–12.5% SDS-PAGE. Antibodies for western blots are as follows: UHRF1 (sc-166898), XRCC4 (sc-8285 and sc-271087), Ku70 (sc-1486), Ku86 (sc-1485), DNA-PKcs (sc-5282), Topo I (sc-5342), and p53 (sc-47698) from Santa Cruz; γH2AX (9718), Topo IIα (4733), cleaved PARP (9541), Puma (D30C10), Phospho-p53 (Ser15) (9286), Phospho-ATM (Ser1981) (5883), ATM (2873), Phospho-ATR (Ser428) (2853), and ATR (2790) from Cell Signaling Technology; tubulin (T6074) and actin (A1978) from Sigma; DNA ligase IV (EPR16531) and Phospho-DNA-PKcs (Ser2056) (ab18192) from abcam; Caspase-3 (40924, Active Motif).

### Quantitative RT-PCR (qPCR)

The qRT-PCR was performed in triplicate using at least five independent sets of cDNA. The results were normalized by the expression level of actin. The primer sequences for XRCC4 are 5'-AATCCACCTTGTTTCTGAACCC-3′ and 5′-CCTTTTTCCATTGCCATGTCATC-3′.

### Immunofluorescence and immunohistochemistry

Y79 control and UHRF1-knockdown cells were plated on poly-d-lysine (PDL)-coated coverslips in 6-well plates the day before chemical treatment. Cells were subjected to pre-treatment with either DMSO or 10 µM ATM inhibitor for 2 h, followed by the treatment with 10 µM etoposide for 2 h without changing media. Then, co-immunostaining was performed with anti-γH2AX (9718, Cell Signaling Technology) and anti-UHRF1 (sc-373750, Santa Cruz) antibodies. Nuclei were counterstained with 4′,6-diamidino-2-phenylindole (DAPI), and cells were visualized with a Zeiss LSM 800 confocal microscope with a 63 × oil objective lens. For immunohistochemistry, paraffin-embedded tissue sections were incubated with anti-XRCC4 (sc-271087, Santa Cruz) or anti-UHRF1 (sc-373750, Santa Cruz) antibody. Immunohistochemistry staining was performed by following the instructions from EliVision DAB detection kit (MAIXIN BIO), followed by nuclear counterstaining with hematoxylin.

### Cell fractionation

Cells were subjected to an acute treatment with high doses of etoposide for 50 min. Then, detergent-soluble cytosolic (S1) and detergent-insoluble chromatin (P2) fractions were collected by following the cell fractionation protocol described previously^39^. Equal amounts of proteins in S1 and P2 fractions were used for immunoblotting. The fractionation quality and loading were monitored by tubulin and histones for the cytosolic and chromatin fractions, respectively. For densitometric analysis for chromatin fractions, histones on the membrane were stained with coomassie blue for normalization of the protein levels in chromatin fractions.

### Plasmid-based NHEJ assay

NHEJ-dependent DSB repair assay was performed by following the procedure described previously^40^ with a few modifications. Briefly, GFP-expression plasmid (pEGFP-C1) was linearized by *Nhe*I/*Age*I digestion to create incompatible ends between the promoter and GFP-coding sequence. The linearized plasmid (10 µg) was transfected into control and UHRF1-knockdown 293T cells (3.5 × 10^6^/dish) with 200 ng of intact RFP-expression vector (pCAG-DsRed) as a transfection control, using X-tremeGENE HP DNA transfection reagent (Roche). At 12 h post-transfection, percentages of GFP- or RFP-positive cells were determined by flow cytometry (FACS), and the relative efficiency of DNA DSB repair was calculated as a ratio of %GFP-positive cells to %RFP-positive cells. Using the same transfectants, DNA was isolated and the end-joining efficiency of the plasmid was determined by qPCR for the ligated junction relative to the uncut GFP-coding sequence.

### Statistical analyses

Statistical significance was determined from at least three independent experiments by two-tailed unpaired student’s *t*-test using GraphPad Prism unless indicated otherwise in the legend.

## Electronic supplementary material

Supplemental material
