# Supplementary material for: UHRF1 depletion sensitizes retinoblastoma cells to chemotherapeutic drugs via downregulation of XRCC4
Source: Cell Death Dis. 2018 Feb 7;9(2):164. doi: 10.1038/s41419-017-0203-4 (PMC5833858; doi:10.1038/s41419-017-0203-4)
Supplement: Supplementary file 1 — Supplemental material [file 41419_2017_203_MOESM1_ESM.pdf]

Supplementary Figures

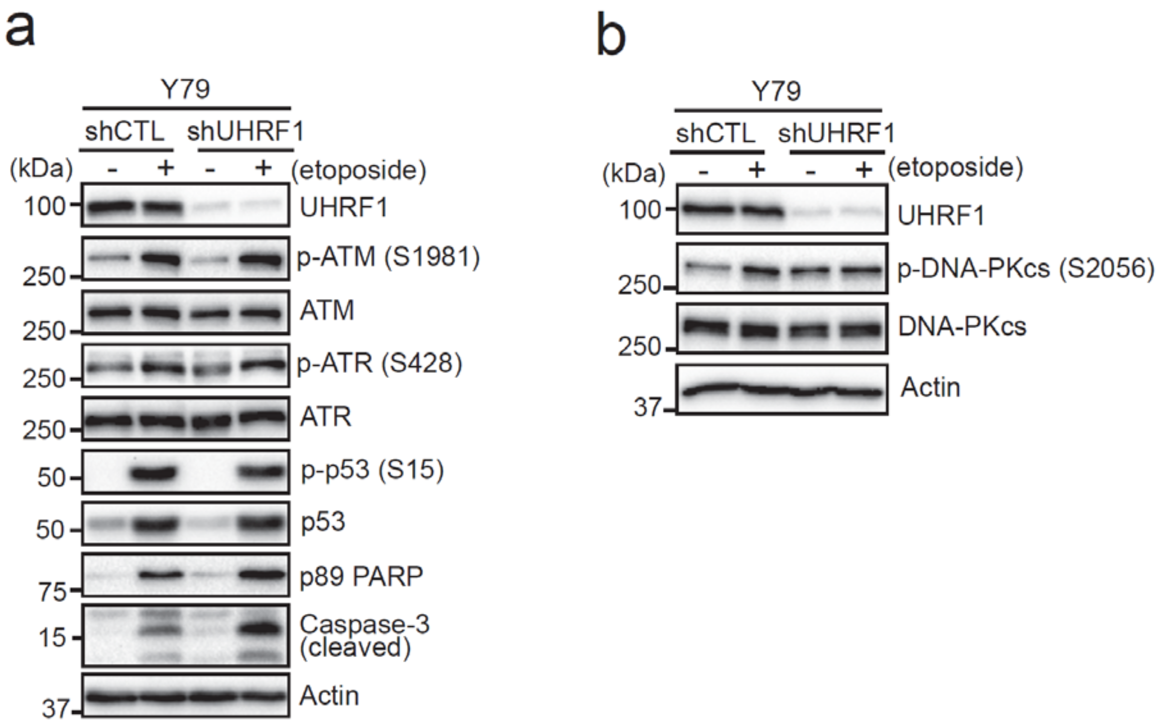

Supplementary Figure S1. Phosphorylation of upstream kinases involved in the DNA damage response. The control and shUHRF1 Y79 cells were treated with either vehicle or 10 µM etoposide for 24 h (**a**) or 2 h (**b**), and the phosphorylation of indicated proteins in response to the etoposide treatment was examined by immunoblotting.

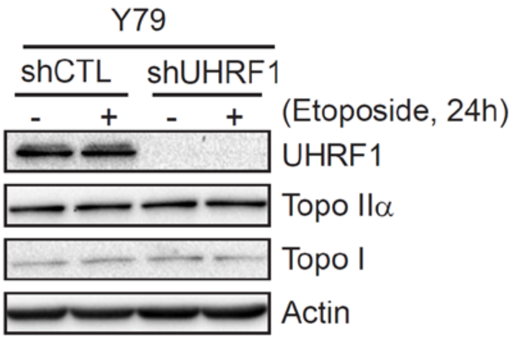

Supplementary Figure S2. Topoisomerase expression in UHRF1-depleted Y79 cells. Immunoblots for topoisomerase I and IIα in control and UHRF1-knockdown Y79 cells treated with vehicle or 10 μM etoposide for 24 h.

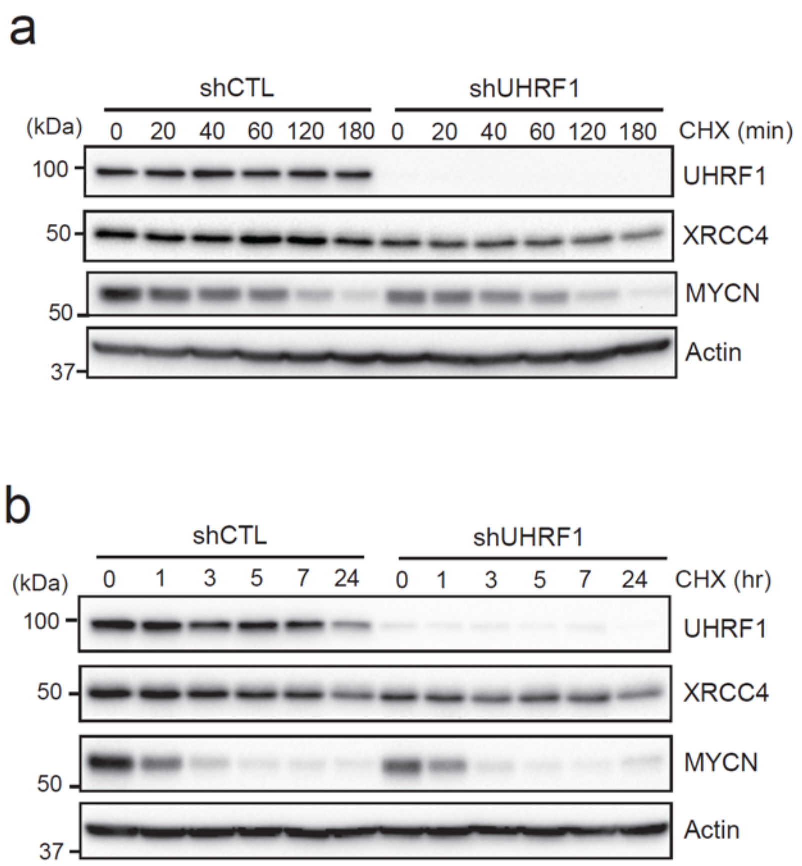

Supplementary Figure S3. XRCC4 protein stability in UHRF1-depleted Y79 cells. The control and shUHRF1 Y79 cells were exposed to cycloheximide (32 µg/ml) for short (**a**) and longer (**b**) treatments as indicated, and the XRCC4 protein level was monitored by immunoblotting. MYCN is shown as a positive control for short-lived proteins.

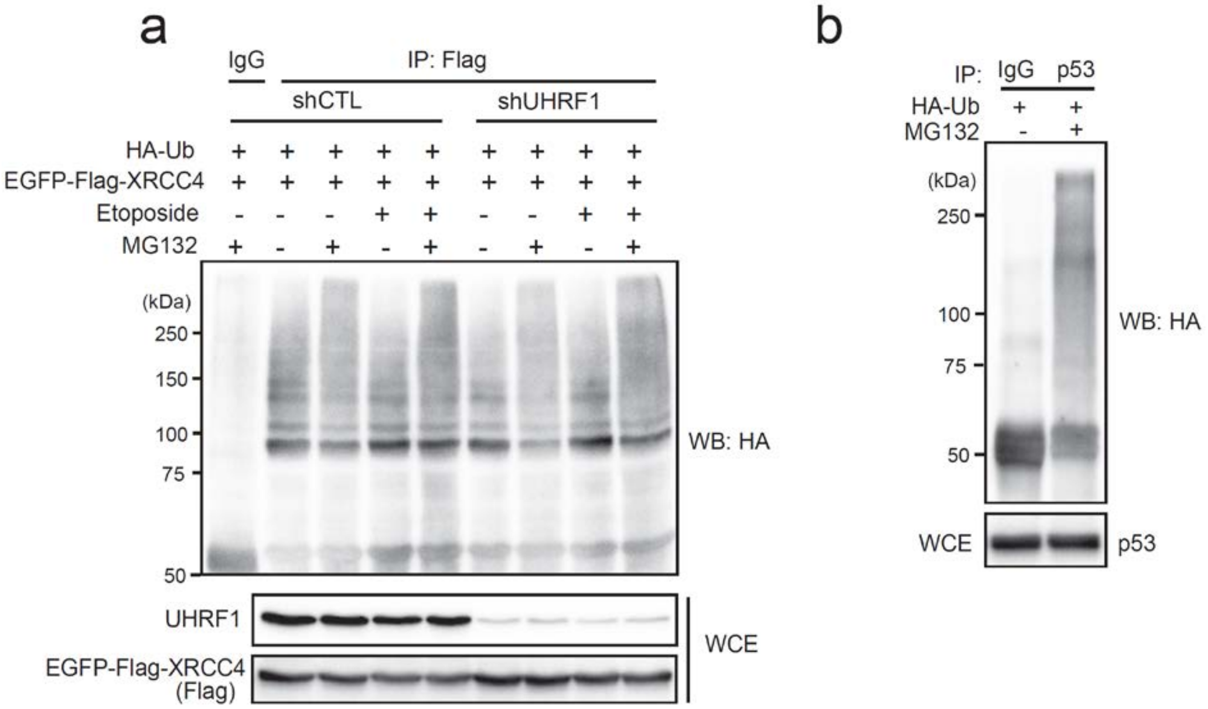

Supplementary Figure S4. Effects of UHRF1 depletion on XRCC4 ubiquitination. (**a**) *In vivo* ubiquitination of XRCC4 in UHRF1-depleted 293T cells in comparison with control-knockdown cells. The stable knockdown cells were co-transfected with HA-Ub and EGFP-Flag-XRCC4 plasmids. At 24 h post-transfection, either vehicle or 10 µM etoposide was added to the culture for 24 h as indicated. After initiation of the 24 h-treatment, 10 µM MG132 or vehicle was added for the last 6 h of the treatment to assess the effects of proteasome inhibition on the XRCC4 ubiquitination in these two knockdown cells. (**b**) Ubiquitination of p53 in 293T cells. Accumulation of HA-ubiquitinated p53 by MG132 treatment (10 µM, 6h) is shown as a positive control for the *in vivo* ubiquitination assay. WCE: whole cell extracts.

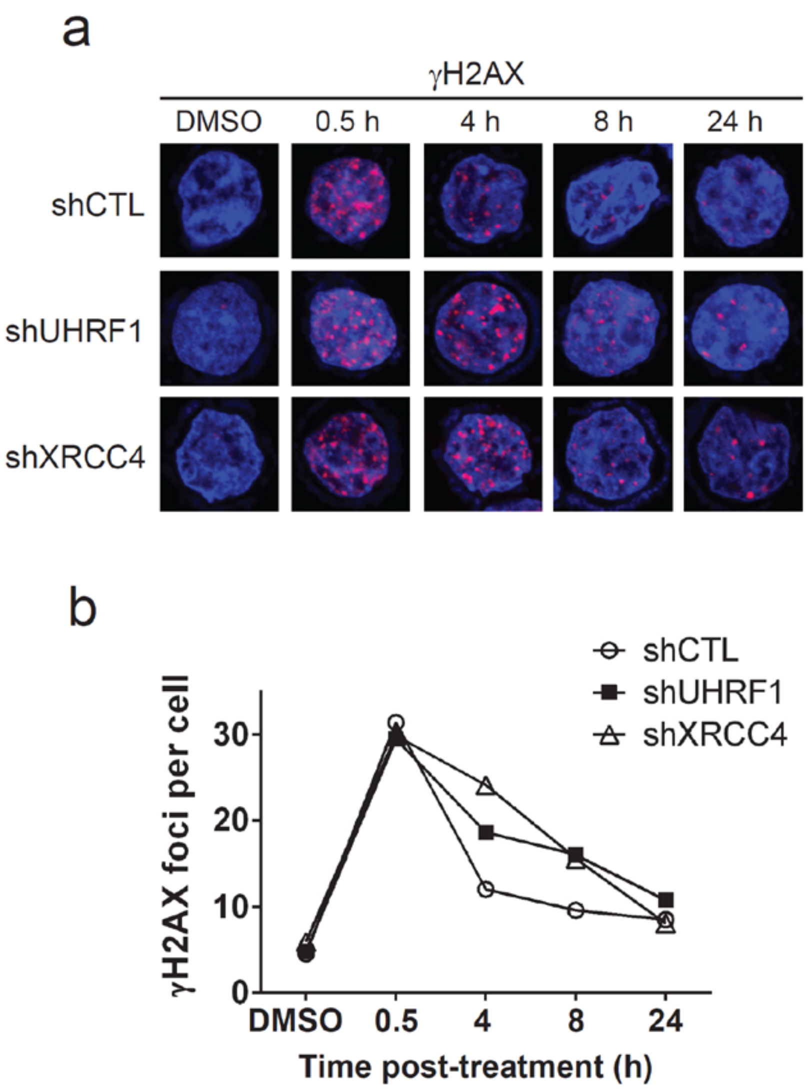

Supplementary Figure S5. Kinetics of γH2AX foci resolution in control, shUHRF1, and shXRCC4 Y79 cells after etoposide-induced DNA damage. Cells were treated with 10 µM etoposide for 1h, and then placed in fresh media without drugs for the indicated time post-treatment. Over 70 total cells at each time point were evaluated for the number of γH2AX foci/cell. (**a**) Representative images of each cell group at the indicated time points. The γH2AX foci marked by red fluorescence are shown in DAPI-stained nuclei. (**b**) Graph showing the mean values of the number of γH2AX foci/cell at each data point.

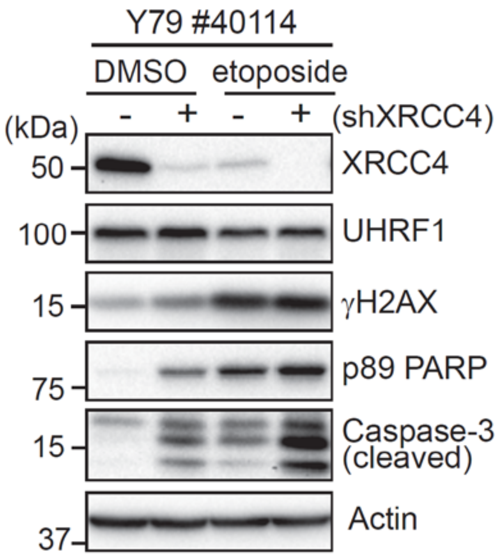

Supplementary Figure S6. XRCC4 knockdown sensitizes retinoblastoma cells to etoposide. Immunoblot analyses are shown for the indicated proteins in shCTL (-) and shXRCC4 (+, clone #40114) Y79 cells after treatment with either vehicle or 10 µM etoposide for 24 h.

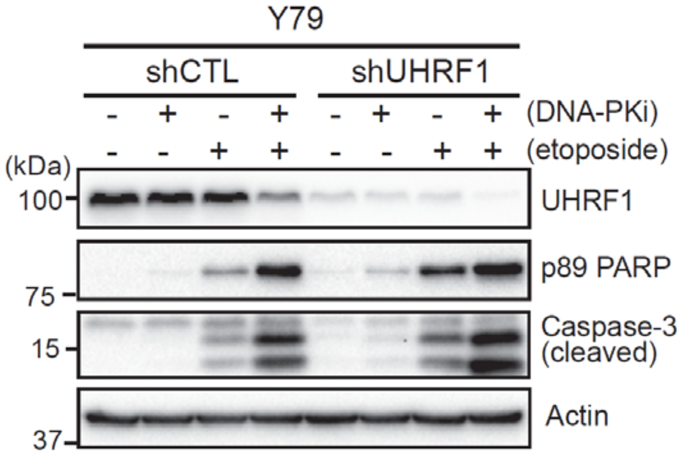

Supplementary Figure S7. Effects of DNA-PK inhibition on apoptosis in response to etoposide. Cells were treated with 10 µM etoposide for 24 h in the presence or absence of 10 µM DNA-PK inhibitor (DNA-PKi, NU7441). Immunoblot analyses are shown for the indicated proteins in shCTL and shUHRF1 Y79 cells.

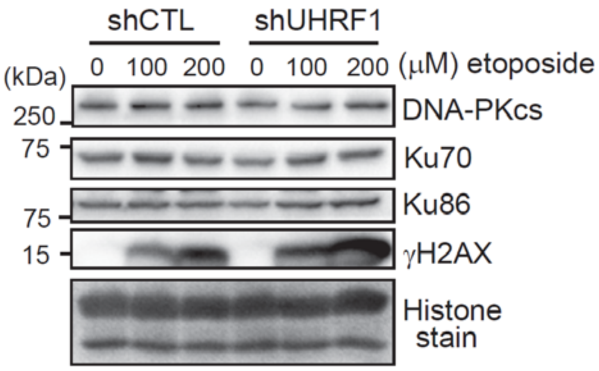

Supplementary Figure S8. Chromatin association of other NHEJ proteins in UHRF1-depleted Y79 cells. Chromatin fractions of control and UHRF1-knockdown Y79 cells were analysed for chromatin association of NHEJ proteins other than DNA ligase IV following the high-dose etoposide treatment for 50 min. Stained histones on the membrane are shown as a loading control.

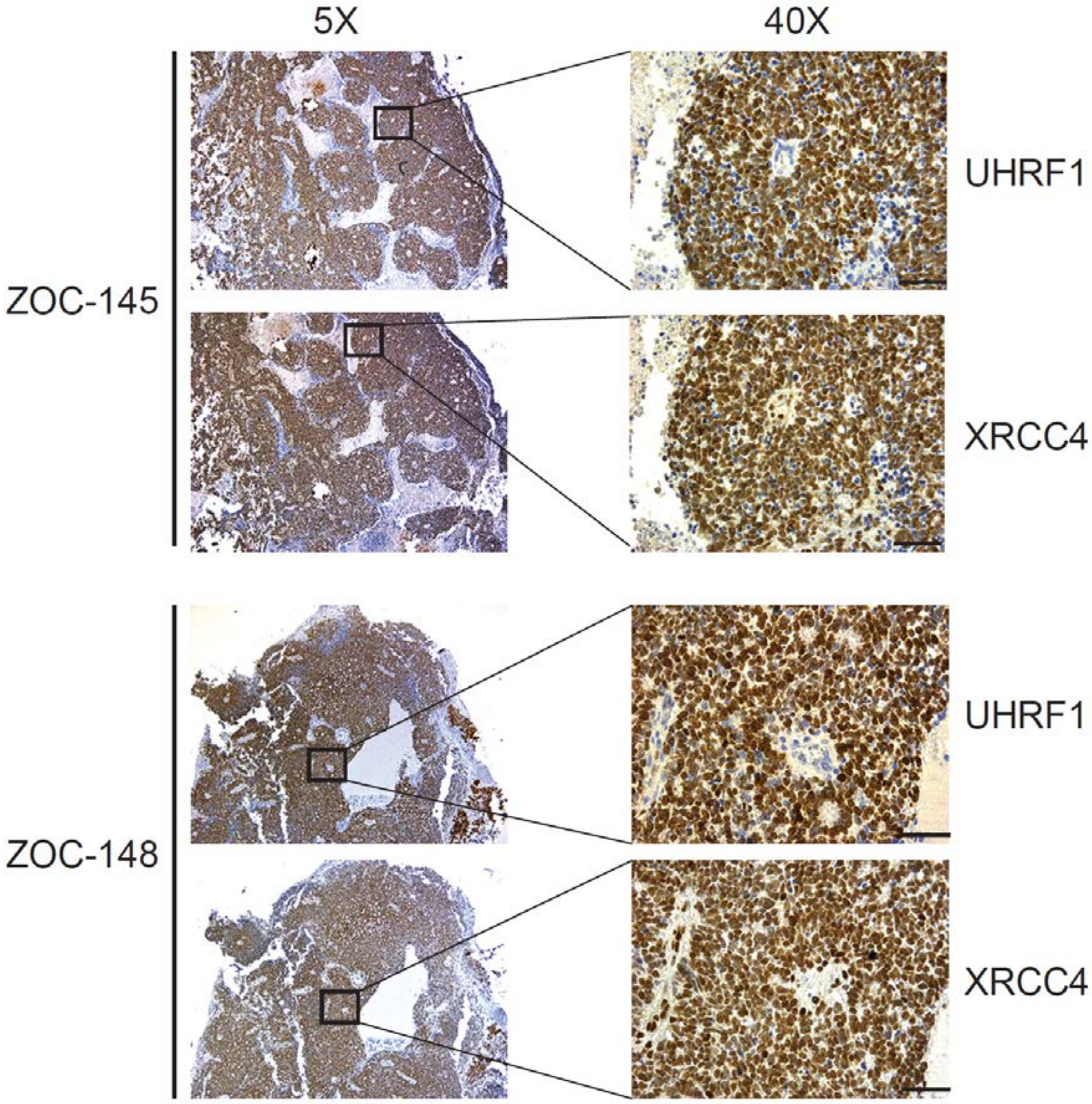

Supplementary Figure S9. Expression of UHRF1 and XRCC4 in human retinoblastoma. Two serial sections of indicated tumor tissues were immunostained for UHRF1 and XRCC4, respectively. The same region of the two serial sections (marked by a square) was magnified to visualize the nuclear staining of UHRF1 and XRCC4 in the tumor. Scale bar: 50 µm.
